# Clinical retrospective analysis with a predictive model for diffused-tenosynovial giant cell tumors of the temporomandibular joint

**DOI:** 10.1186/s12885-023-11587-7

**Published:** 2023-11-03

**Authors:** Ying Liu, Yingying Huang, Dongwang Zhu, Jiang Li, Tongchao Zhao, Yining He, Ronghui Xia, Laiping Zhong

**Affiliations:** 1 Department of Oral & Maxillofacial Head & Neck Oncology, Ninth People’s Hospital, College of Stomatology, Shanghai Jiao Tong University School of Medicine, Shanghai, China; 2 Department of Oral Pathology, Ninth People’s Hospital, College of Stomatology, Shanghai Jiao Tong University School of Medicine, Shanghai, China; 3 Biostatistics Office of Clinical Research Unit, Shanghai Ninth People’s Hospital, Shanghai JiaoTong University School of Medicine, Shanghai, China; 4 Department of Stomatology Oral maxillofacial Head and Neck Surgery, Huashan Hospital, Fudan University, Shanghai, China; 5 Department of Pathology and Laboratory Medicine, Tulane University School of Medicine, New Orleans, LA USA

**Keywords:** Diffused-tenosynovial giant cell Tumor, Temporomandibular joint, Local recurrence-free survival, Ki-67 index, Colony-stimulating factor 1 receptor

## Abstract

**Background:**

This study aimed to find out the characteristics in relation to tumor recurrence in diffused-tenosynovial giant cell tumor of temporomandibular joint and to develop and validate the prognostic model for personalized prediction.

**Methods:**

From April 2009 to January 2021, patients with diffused-tenosynovial giant cell tumor of temporomandibular joint at a single center were included in this study. The clinical features and local recurrence-free survival were assessed through the expression of the Ki-67 index and colony-stimulating factor 1 receptor expression. Both univariate and multivariate analyses were performed on the prognostic factors for local recurrence-free survival. An independent predictor nomogram and pertinent tumor characteristics were included.

**Results:**

The retrospective study enrolling seventy eligible patients at the Ninth People’s Hospital, Shanghai Jiao Tong University School of Medicine. During the follow-up time, eleven patients suffered tumor recurrence. Age was an independent risk factor for local recurrence-free survival (P = 0.032). The Ki-67 index varied significantly in different sites (P = 0.034) and tumor volume (P = 0.017). Multivariate logistic regression was used to develop the prediction model using both statistical significance and prognostic indicators. The C-index of the nomogram based on age, site, Ki-67, and colony-stimulating factor 1 receptor was 0.833. These variates provided good predicted accuracy for a nomogram on local recurrence-free survival. Diffused-tenosynovial giant cell tumor from the temporomandibular joint is extremely uncommon, and certain clinical traits are linked to the tumor proliferation index.

**Conclusions:**

We identified the risk indicators and developed a nomogram in this study to forecast the likelihood of local recurrence-free survival in patients with diffused-tenosynovial giant cell tumor from temporomandibular joint.

**Supplementary Information:**

The online version contains supplementary material available at 10.1186/s12885-023-11587-7.

## Introduction

Tendon Sheath of Giant Cell Tumor (TSGCT), also known as Pigmented Villonodular Synovitis (PVNS), is an uncommon benign soft tissue tumor that can cause joint or limb swelling, discomfort, stiffness, and restricted mobility [1]. TSGCT is thought to affect 1 in 1,800,000 people annually [2]. The onset age is between 20 and 50 years old, and there are twice as many women as men documented in the literature [3]. TSGCT are broadly categorized as disseminated and localized types. Knees, hips, and finger joints are more frequently affected by Diffused Tendon Sheath of Giant Cell Tumor (D-TSGCT) lesions than temporomandibular joints(TMJ), where little over 100 instances have been documented. Around 80% of patients reportedly presented with symptoms, according to relevant literature [4]. Meanwhile, D-TSGCT that develops in the TMJ region typically involves bone loss and invades nearby structures [3, 5]. In T2 weighted pictures with low signal intensity, the MR imaging of TSGCT exhibits a typical “blooming effect,“ which is useful for clinical diagnosis [6]. The conventional course of care for a large cell tumor of the tendon sheath in the TMJ is surgical resection. For cases where the TMJ treatment left ambiguous margins or persistent malignancies, post-operative radiation was used [7]. After complete resection, the recurrence rate for D-TSGCT is between 20 and 50%, with a TMJ recurrence rate of 14%.^2,9–11^

Tenosynovial giant cell tumors are benign, proliferative, and destructive lesions of the synovium that originate in joints, tendon sheaths, and bursae, according to the World Health Organization’s (WHO) classification of soft tissue and bone cancers [12]. According to Rao et al., these lesions were brought on by aberrant fibroblast and histiocyte growth [7]. Furthermore, West et al. have suggested that these lesions are brought on by the gene translocation-induced attraction of multinucleated giant cells to tumor cells [8]. Macrophages and colony-stimulating factor 1 receptor (CSF1R) dependent inflammatory cells are components of TSGCT, which may be controlled by tumor cells and result in bone resorption. CSF-1R inhibitor was found to have positive effects in clinical trials and was subsequently licensed by the FDA for the treatment of TSGCT [9–11]. The biological behavior and growth pattern of D-TSGCT in the TMJ area are not clear. No studies were identified that directly investigated this issue in TMJ D-TSGCT. Ki-67 is a reliable marker for malignant tumor proliferation, and the results of detection are accurate. In diffused-type, large-volume, and recurrent TSGCT patients, increased levels of Ki-67 expression were documented in earlier research [12, 13]. Wang et al. provides the first analysis of Ki-67 in diffuse-type TSGCTs of the TMJ, demonstrating significantly higher Ki-67 indices in recurrent versus non-recurrent TMJ tumors [14]. However, the combined expression of the colony stimulating factor 1 receptor (CSF1R) mutation and Ki-67 is lacking evidence in D-TSGCT of the TMJ, representing an area requiring further study. These biomarkers may have also an association with tumor invasion and have the potential clinical applicability in the estimation of recurrence. The prognosis variables for patients with D-TSGCT are not fully understood at the moment. In the present study, we focused on the clinical characteristics, Ki-67 expression, CSF1R expression, and their interrelationship in a group of D-TSGCT cases affecting the TMJ. Owing to the lack of a practical predictive method, we established the first nomogram for predicting the recurrence of D-TSGCT of TMJ. Nomograms are statistical tools to generate individualized predictions of a clinical outcome based on a combination of factors. Their use is increasing in healthcare for risk stratification. Since the related studies are case reports and it is hard to build a nomogram for limited cases [15, 16].

## Materials and methods

### Data collection and study population

This study was approved by the Ethical Committee of Ninth People’s Hospital, Shanghai Jiao Tong University School of Medicine (Shanghai, China) (SH9H-2022-T298-1). Informed consent for participating in the clinical study was signed by all involved patients. From April 2009 to Jan 2021, a total of 70 cases of D-TSGCT in the TMJ area were diagnosed and treated in our hospital, their paraffin specimens and clinical characteristics such as sex, age, site, symptom, size, bone destruction, surgery complication and follow-up time were collected (Table 1 and Supplementary Table 1). All patients underwent wide local excision aiming for negative surgical margins. Positive margins were re-excised if feasible to obtain clear margins. Patients were followed every 6 months for the first 2 years, then annually for years 3–5, with CT or MRI performed at these visits to monitor for recurrence.

**Table 1:** Clinical characteristics of D-TSGCT patients from temporomandibular joint

Variable	Number (n)	Percentage(%)
Total	70	100%
**Age(years)**		
<46	33	47.1
≥ 46	37	52.9
**Sex**		
Male	39	55.7
Female	31	44.3
**Tumor size**		
<2.5 cm	20	28.6
≥ 2.5 cm	50	71.4
**Tumor location**		
Supra-articular cavity	48	68.6
Subarticular cavity	22	31.4
**Bone invasion**		
Yes	61	87.1
No	9	12.9
**Smoking history**		
Yes	9	12.9
No	61	87.1
**Drinking history**		
Yes	6	8.6
No	64	91.4
**Post-operative complication**		
Yes	24	34.3
No	46	65.7
**Ki-67 index**		
Low	32	45.7
High	38	54.3
**CSF-1R expression**		
Low	16	22.9
High	54	77.1

### Immunohistochemistry and immunofluorescence test

Immunohistochemical staining for Ki-67 and CSF-1R was carried out using standard immunohistochemistry protocols widely employed in literature [17]. In brief, 4 μm thick sections were used for immunohistochemical staining. After deparaffinization, peroxidase block, and heat-induced epitope retrieval, primary mouse monoclonal Ki-67antibody (MAB-0672) at 1:400 dilution and rabbit polyclonal CSF-1R antibody (LifespanBiosciences, USA) at dilution 1:500 was added overnight at 4 °C, then visualized using 3,30-diaminobenzidine (DAB) detection kit (DakoCytomation, Glostrup, Denmark) containing goat secondary antibody molecules against mouse and rabbit immunoglobulin and DAB chromogen. Negative control was performed using a phosphate buffer solution (PBS) instead of a primary antibody. The fluorescent secondary antibody was goat anti-rabbit IgG Cy3 (Servicebio, Wuhan). Microscopic examination was performed by two pathologists and all samples were blinded.

Ki-67 immunohistochemical evaluation criteria: the brown-yellow particles on the cell nucleus are judged as positive, regardless of the staining intensity, all brown-stained cell nuclei are counted as positive. Two senior pathologists used a double-blind method to read and analyze the film. According to the results of immunohistochemistry, 5 fields were randomly selected under a high-power microscope, and 100 cells were counted in each field. Record the ratio of positive cells.

CSF-1R/CSF-1 immunohistochemical scoring standard: The immune response score is the product of the staining intensity (SI) and the percentage of positive cells (PP), that is, IRS = SI*PP. The positive rate score is calculated as the percentage of the number of positive cells: 0 = negative, < 5%; 1 = weakly positive, 5%~24%; 2 = moderately positive, 25%~49%; 3 = strongly positive, 50%~74%; 4 = super positive, ≥ 75%; grade of staining intensity: 0 = none; 1 = weak positive; 2 = positive; 3 = strong positive [18].

An immunofluorescence test was used to localize the Ki-67 and CSF-1R protein expression. Image J was applied to detect the fluorescence intensity of each protein [19]. Three fields were randomly selected with caseviewer 3.3 under a high-power microscope.

### Statistics

SPSS statistical software version 23.0 (IBM Corp) and R software version 6.3 (R Project forStatistical Computing) was used for all statistical analyses. Wilcoxon x2 test and student t test was used. local recurrence-free survival (LRFS) is defined as the time from the first treatment of the tumor to the local tumor recurrence, and the recurrence is defined as the tumor after the complete resection of the tumor through imaging or clinical examination. LRFS rates were analyzed using the Kaplan-Meier method, and inter-subgroup differences were examined by the log-rank test. The best cut-off value of Ki-67 and CSF1R expression level was determined based on the receiver operating characteristic (ROC) curve to find the boundary value and divide the variables into binary variables. P values were 2-sided and were considered statistically significant if less than 0.05. In order to build the nomogram for the prediction of the recurrence, a statistical significance level of 0.1 in cox univariate analysis was selected as a risk factor. We aimed to establish a model for the individualized estimation of LRFS in D-TSGCT. Nomograms to estimate 3-year and 5-year LRFS were generated based on the risk factors established by multivariate analysis and immunohistochemistry indicators with R version 6.3. The performance of the nomogram to discriminate and calibrate depended on the Harrell concordance index (C index) and calibration curve. The value of the C index ranged from 0.5 to 1.0 and a value of that at least 0.7 indicated that the model has a good discriminating ability. Decision curve analysis (DCA) was employed to examine the clinical net benefit of a predictive model by the rmda(regularized multivariate data analysis) package in R. It is an R package for regularized classification and feature selection methods [20].

## Results

### Patient demographic characteristics

**Fig. 1:**
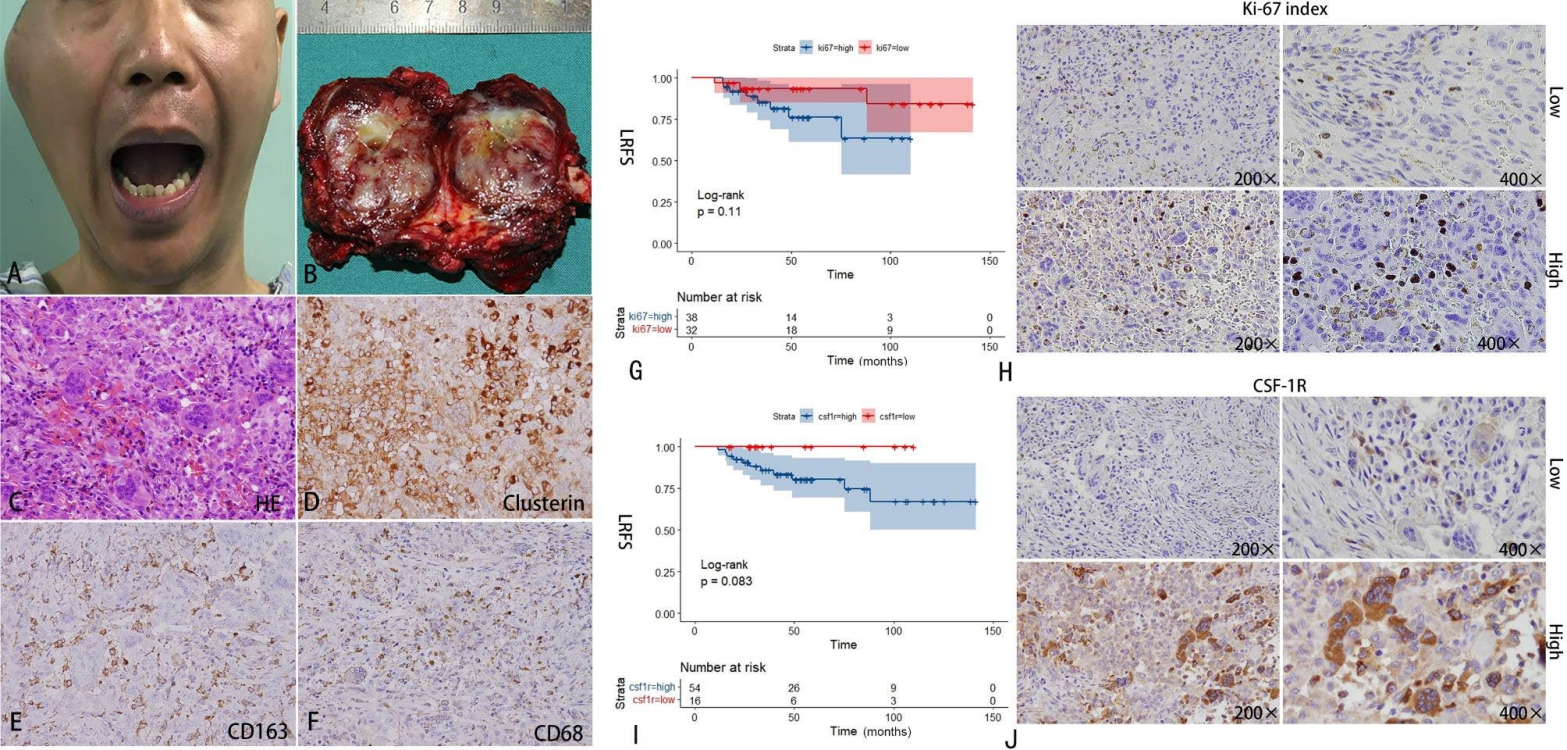
Clinical and microscopic features of a diffused-tenosynovial giant cell tumor (D-TSGCT) in the temporal-mandibular joint (TMJ) displaying a preauricular mass on the right side of the TMJ area (A); The tumor of the section surface is a multinodular yellow, white, and brown tissue but does not clearly display a capsule(B);Haematoxylin and eosin (H&E400×)staining shows histiocytes, synovium-like monocytes and multibucleated giant cells(C); The synovium-like monocytes expressed clusterin(D); The small histiocytoid cells expressed CD163(E); The multinucleated giant cells and a few small histiocytoid cells expressed CD68(F);Local recurrence-free survival (LRFS) time and Kaplan-Meier survival curve for various Ki-67 grades(G); Representative images of different Ki-67 degrees, from low power 200× to high power400×(H); Local recurrence-free survival (LRFS) time and Kaplan-Meier survival curve for various colony stimulating factor1 receptor grades(I); Representative images of different colony stimulating factor1 receptor degrees, from low power 200× to high power400×(J)

Between April 2009 to Jan 2021, a total of 70 patients with D-TSGCT in the TMJ area underwent radical surgery in our hospital. A summary of basic demography is presented in Table 1. Detailed information was recorded in Table S1. There were 31 (44.3%) females and 39 (55.7%) males, with a gender ratio of 1.26:1. The median age was 46 years, with a range of ages from 23 to 72 years. Headache (45.7%), limited mouth opening (17.1%), hearing loss (22.9%), and slowly swelling pre-auricular mass (22.9%) (Fig. 1A) were the most frequent complaints. Based on the analysis of the image, the tumor’s average diameter was 3.2 cm with a range of 1.3 cm. According to an MRI/CT imaging, 48 patients (68.6%) exhibited basal skull basal bone degeneration, and 31 patients (44.3%) had condyle invasion at the time of the initial diagnosis. The infratemporal fossa of the skull base was restored in 48 patients (68.6%), with the major restoration methods including bone flap repair, soft tissue repair, and titanium mesh repair. There were 15 patients (21.4%) who underwent condyle repair, including 6 patients who had rib repair and 9 patients who needed replacing of an artificial joint.

All the patients received surgical treatment. Twenty-four patients (34.3%) had post-operative complications. Facial paralysis (14.3%), limited mouth opening (5.7%), malocclusion (4.3%), and hearing loss (4.3%) were the four main post-operative complications. The median follow-up time was 51.3 months (ranging from 11.6 ~ 141.4 months). Patients younger than 46 years (the median age) had an average follow-up of 53.83 months (median 41.9 months), while patients 46 years or older had a mean follow-up of 54.69 months (median 50.8 months). No one received adjuvant treatment after surgery. A total of eleven patients showed recurrence and the average recurrence time was 36.3 months. All of the patients were still alive at the last follow-up date, and only one patient underwent salvage surgery. Complete surgical resection with negative margins was achieved in all patients except one (patient 36), who underwent re-excision to obtain negative margins after initial positive margins were identified upon pathological analysis. Detailed pathological margin status for each patient is provided in Supplementary Table 1.

### Histopathological characteristics

The surface of the tumor was either dark red, reddish brown, or gray with or without cystic alteration (Fig. 1B). The lesion was revealed to be made up of synovium-like monocytes, tiny histiocytoid cells, and multinucleated large cells under a high magnification microscope, with varying proportions of each cell. Various degrees of background hemosiderosis flavin deposition and scattered lymphocyte infiltration were visible. All patients had synovium-like monocytes that produced clusterin, and the cytoplasm of these cells stained positively. All small histiocytoid cells exhibited CD163, and several also expressed CD68, with positive staining in the cytoplasm. CD68 was expressed in multinucleated giant cells. (Fig. 1C-F) The expression level of the Ki-67 index was significantly related to site and tumor size.

According to the ROC curve, at a cut-off value of 2%, the intensity of the Ki-67 gene was divided into two groups: 32 samples with low expression, which accounted for 45.7% (32/70), and 38 samples with high expression, which accounted for 54.3% (38/70). Figure 1 H displayed the LRFS between various levels of Ki-67 expression. The LRFS between the different expression levels of Ki-67 was shown in Fig. 1G.

The positive expression of Ki-67 in patients with supra-articular cavity was significantly higher than that in patients with a subarticular cavity, and there was a significant difference in the expression of Ki-67 between the two sites (super-articular cavity vs. subarticular cavity; P = 0.034). In patients with different tumor volumes (diameter ≥ 2.5 cm vs. diameter < 2.5 cm), there was a substantially varied expression of Ki-67 (P = 0.017). Patients with tumors that were less than 2.5 cm in diameter had significantly higher levels of positive Ki-67 expression than patients whose tumors were larger than 2.5 cm.

### The expression level of CSF-1R was consistent with the expression of the Ki-67 index

CSF-1R expression intensity was separated at the cut-off value of 3% into two groups: 16 samples with low expression, accounting for 22.9% (16/70), and 38 samples with high expression, accounting for 77.1% (54/70). The LRFS between different expressions of CSF1R was shown in Fig. 1I. No significant correlation was found between the positive expression of CSF-1R and clinical features. Detailed information on Ki-67 expression and CSF-1R expression in D-TSGCT patients was listed in Table 2.

**Table 2:** The Ki-67 index and CSF-1R expression in seventy patients diagnosed with D-TSGCT from TMJ

Variable	N = 70	Ki67 index(%)		CSF-1R expression(%)	
			P value*		P value*
	n(%)	Mean ± SD		Mean ± SD	
**Age(years)**					
<46	33(47.1)	4.4 ± 4.0		4.6 ± 1.6	
≥ 46	37(52.9)	3.6 ± 4.0	0.396	4.4 ± 1.7	0.554
**Sex**					
Male	39(55.7)	4.2 ± 4.2		4.5 ± 1.5	
Female	31(44.3)	3.6 ± 4.0	0.568	4.5 ± 1.7	0.993
**Tumor size**					
<2.5 cm	20(28.6)	2.2 ± 1.5		4.7 ± 1.4	
≥ 2.5 cm	50(71.4)	4.7 ± 4.5	0.017	4.4 ± 1.7	0.546
**Tumor location**					
Supra-articular cavity	48(68.6)	4.6 ± 4.5		4.3 ± 1.6	
Subarticular cavity	22(31.4)	2.5 ± 2.2	0.034	4.8 ± 1.6	0.222
**Bone invasion**					
Yes	61(68.6)	4.0 ± 4.2		4.5 ± 1.6	
No	9(31.4)	3.3 ± 3.0	0.623	4.6 ± 1.9	0.935
**Smoking history**					
Yes	9(12.9)	3.7 ± 3.3		5.1 ± 1.1	
No	61(87.1)	4.0 ± 4.2	0.819	4.4 ± 1.7	0.237
**Drinking history**					
Yes	6(8.6)	2.7 ± 3.6		4.8 ± 0.9	
No	64(91.4)	4.1 ± 4.1	0.416	4.5 ± 1.7	0.616
**Post-operative complication**					
Yes	24(34.3)	4.5 ± 4.7		4.2 ± 1.7	
No	46(65.7)	3.6 ± 3.7	0.42	4.7 ± 1.5	0.195

**Fig. 2:**
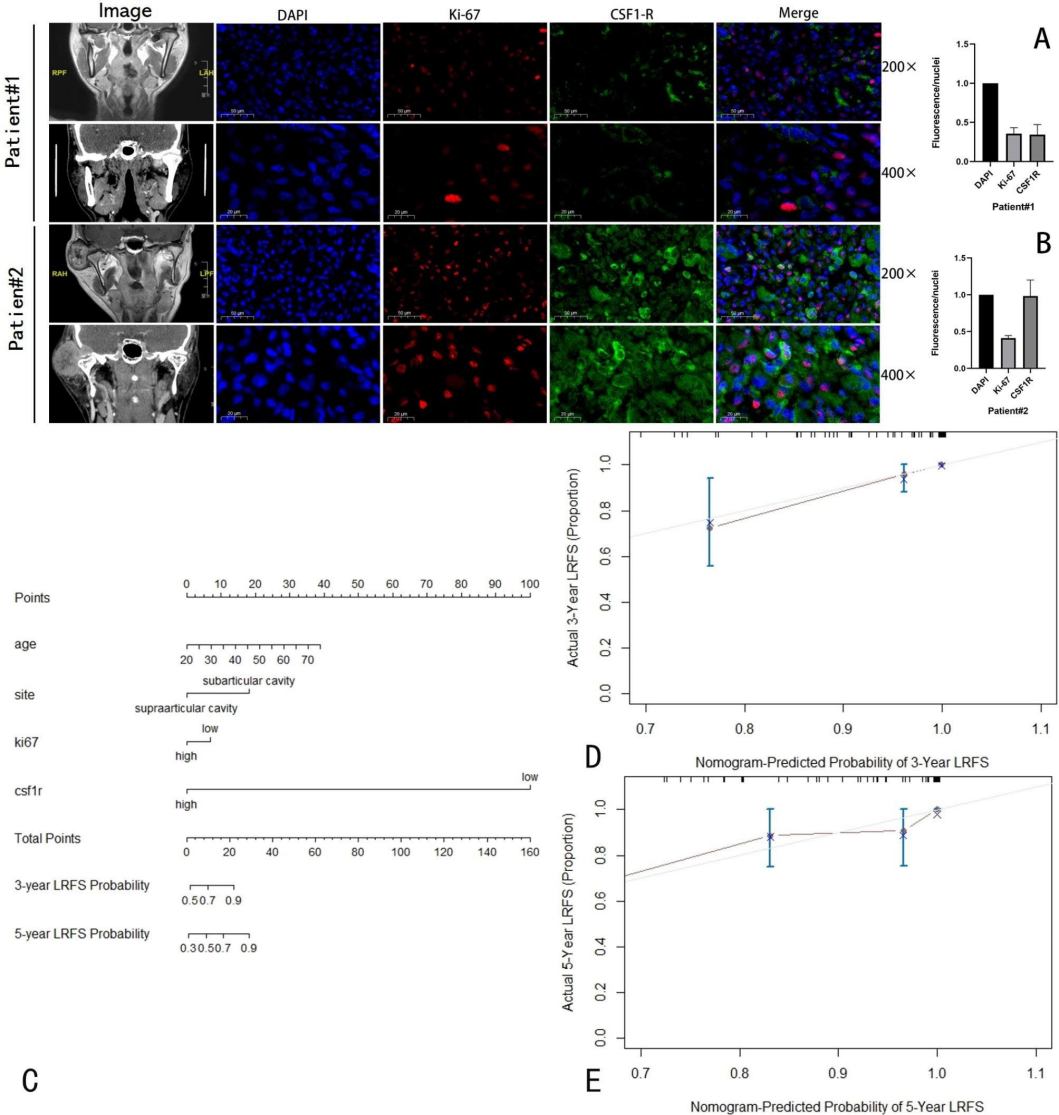
Typical case 1: T2-weighed sagittal MRI and CT image of D-TSGCT on the left side of TMJ with small volume near the subarticular disc(A); Typical case 2: MRI-scan and CT image of D-TSGCT on the right side of TMJ display a larger supra-articular component and bone erosion(B); The D-TSGCT of the two patients is displayed in conjunction with various magnificence CSF-1R and Ki-67 immunofluorescence staining. Nuclear DAPI staining is blue fluorescence, Ki-67 staining is red fluorescence, and CSF-1R is green fluorescence of multinucleated large cells; Nomogram predicting the prognosis for D-TSGCT patients in TMJ location(C); Calibration plots for the predicted and observed local recurrence free survival at D, 3 years; E, 5 years. The x-axis demonstrates the nomogram-predicted probability, and the y-axis shows the actual observed probability

Mononuclear histiocytes, multinucleated giant cells, and big synovial monocytes make up the majority of the tumor structure. Ki-67 was primarily expressed in the nucleus of monocytes. The cytoplasm and cell membrane surfaces of monocytes and macrophages were the primary sites of CSF-1R expression. The different expressions of Ki-67 and CSF-1R with immunohistochemical staining from TMJ D-TSGCT samples were presented in Fig. 1H and J. In the immunofluorescence labeling of the example tissues, CSF-1R expression tended to increase as the Ki-67 index rose. But in the two represented samples, there was no discernible difference in the ratio of the target protein to DAPI in Fig. 2A,B.

### Nomogram model for prediction on LRFS in D-TSGCT of TMJ

Age, site, Ki-67 index, and CSF-1R variables were included in the model based on the findings of the Cox regression and associated clinical values (The detailed information was listed in Tables 3 and 4). By the variables examined, a nomogram for LRFS prediction was created. Utilizing calibration curves and C-index values, nomogram validity was evaluated. The C-index value was 0.815 [95% confidence interval (CI) = 0.69–0.94], indicating favorable discrimination by the nomogram (Fig. 2C). The calibration curves of the nomogram showed high consistencies between the predicted and observed LRFS probability (Fig. 2D,E). The decision curve analysis for the nomogram model was depicted in Figure S1. In conclusion, the nomogram for LRFS prediction possessed strong calibrating and discriminative capabilities.

**Table 3:** Univariate analysis for the estimation of risk factors for LRFS and nomogram model

Variable		Hazard ratio	95%CI		P value
Age	3.25		0.86–12.3	0.07	
Sex	1.38		0.40–4.47	0.61	
Site	4.49		0.57–35.17	0.12	
Size	4.39		0.56–34.33	0.16	
Bone invasion	24.48		0.00-142447	0.47	
Smoking	1.6		0.20-12.54	0.65	
Drinking	0.98		0.13–7.99	0.99	
Post-operative complication	0.84		0.22–3.21	0.80	
Ki-67 index	0.34		0.09–1.33	0.11	
CSF-1R	0.03		0.00-16.66	0.08	

**Table 4:** Multivariate analysis for the estimation of risk factors for LRFS and nomogram model

Variable		Hazard ratio	95%CI	P value
Age	10.18		1.04–15.40	0.03
Site	0.93		0.02–1.08	0.04
Ki-67 index	0.96		0.82–1.14	0.65
CSF-1R	1.45		0.84–2.52	0.19

## Discussion

D-TSGCT from the TMJ location was a relatively rare tumor and the biological character and the prognosis were not clear because of little clinical evidence. Our findings indicated that young adults were primarily impacted by D-TSGCT caused by TMJ. Age was an independent predictor of recurrence. The median follow-up was shorter in the younger group (41.9 months) versus the older group (50.8 months). Age remained a significant predictor of recurrence on multivariate analysis, indicating it represents an intrinsic biological driver of recurrent disease, rather than just a reflection of observation time. Younger age may confer inherent tumor traits leading to increased proliferative/invasive potential and recurrent growth. The underlying biological mechanisms for this warrant further investigation but are beyond the scope of this clinical outcomes study. Even though D-TSGCT was a benign tumor with an invasion of proliferative and inflammatory cells, roughly 87.1% of tumors showed bone damage. Previous studies reported that about 30%~50% of cases had skull base invasion. The 68.6% rate of middle cranial fossa invasion in my cohort closely matches the 66% rate found in the systematic review by Kanbour et al. This aligns with their large case series and confirms the high propensity for intracranial extension in TMJ D-TSGCT [21]. The skull base (48/70) and condyle (31/70) were the most affected bone in the TMJ area. We found in this study that the tumor site probably had a relationship with various types of bone damage. Tumors from the supra-articular cavity prone to have skull base invasion while tumors from the sub-articular cavity prone to have condyle invasion. While we observed a mild male predominance, the relevance of this finding is questionable given the limited sample size and typically small case series comprising the current TMJ D-TSGCT literature. A recent systematic review by Kanbour et al. analyzed 63 published cases of D-TSGCT affecting the temporomandibular joint, finding a male prevalence of 57.1%.^21^ Larger epidemiologic studies have not established gender as a risk factor at any site. Uncontrolled demographic factors and referral biases likely explain the variance from an expected female preponderance. Definitive evidence linking gender specifically to TMJ D-TSGCT propensity is lacking, thus it is premature to conclude the gender ratio fundamentally differs without additional investigation controlling for confounders in larger, multi-center cohorts. Currently, it remains unclear if gender truly impacts TMJ D-TSGCT susceptibility compared to other locations.

Our results also showed that the expression of Ki-67 from the supra-articular cavity is higher than the expression of Ki-67 in sub-articular space lesions (P = 0.032). The supra-articular cavity tumor was larger and more likely to recur (79.2%). In the study of Wang et al., it was found that the average Ki-67 index of relapsed cases was significantly higher than that of non-relapsed cases (P < 0.05). Adjuvant radiotherapy is used to control local tumor remnants or recurrent tumors, and it is believed that the Ki-67 index has predictive significance for recurrence [14]. Relapsed cases in this study exhibited a substantially higher expression of Ki-67 than non-relapsed cases, although there was no discernible difference in Ki-67 levels between the two groups. Furthermore, there was a strong correlation between tumor size and the expression of Ki-67. The expression of Ki-67 was higher in tumors with a diameter of more than 2.5 cm. Ki-67 is a marker that reflects the proliferation of malignant tumors, which could reflect the proliferation activity of cells. The malignant transformation of diffuse giant cell tumors of tendon sheath is rare, and it is classified as a rare sarcoma. Li et al. found the medium volume of the malignant D-TSGCT is larger (P = 0.036) and Ki-67 expression is higher (P < 0.001) compared with benign D-TSGCT. According to Weckauf et al., the expression level of Ki-67 increased in recurring tumors and was higher in diffuse giant cell tumors of the tendon sheath than in localized giant cell tumors of the tendon sheath [13]. Similarly, our previous results also confirmed that tumors originating from the supra-articular space were larger, more aggressive and more likely to recur [22].

Specific gene translocations, which led to the recruitment of CSF-1R-expressing cells such as monocytes, macrophages, and some tumor cells, were the primary cause of TSGCT. It was reported that the expression of CSF-1R was associated with poor prognosis in certain cancers [23]. Tumor associate macrophages (TAM) are associated with poor prognosis in most solid tumors, which may represent future anti-cancer potential targets for treatment.^24^ As an important tyrosine kinase transmembrane receptor on the surface of TAM cells, CSF-1R regulates the development, morphology, survival and function of TAM. Although the expression of CSF-1R was not significantly related to a variety of clinical features in D-TSGCT, there was a tendency that expression of CSF-1R was consistent with the expression of Ki-67. Patients with higher CSF-1R expression were more likely to recur. The activation of the CSF-1/CSF-1R pathway could contribute to the conversion of TAMs from M1 to M2 phenotype [24]. The function of M2 type macrophages had immunosuppression and promoted tumor growth and macrophages that appeared in tumors were mainly M2 phenotype [25, 26]. We might deduce that the poor prognosis in D-TSGCT is caused by the high expression of CSF1R, which may result in M2 macrophage aggregation.

As was mentioned before, D-TSGCT originated from TMJ and had specific clinical features. D-TSGCT was classified as a borderline tumor by the ICD WHO and was vulnerable to recurrence following excision. As a result, this nomogram performed well in terms of recurrence prediction when using the C-index, calibration curve, and DCA based on the TRIPOD statement standard. Our study suggests that pre-operative factors, including age, tumor growth invasion, and higher Ki-67 expression level are associated with tumor recurrence. A recurrence rate of 21% within the first 5 years following treatments was seen in a meta-analysis of D-TSGCT in foot and ankle lesions [27]. A higher recurrence rate of 42.8% after treatment of the primary tumor was confirmed from a multinational, multicenter observational study in D-TSGCT [28]. Retrospective investigation of TMJ D-TSGCT patients by Carlson revealed an 11% local recurrence incidence in surgically treated patients [29]. Our recurrence rate was 9.5%, identical to the 9.52% recurrence rate in the 63 reported TMJ D-TSGCT reviewed cases [21]. In our earlier investigation, the average time for D-TSGCT in TMJ to recur in published references was roughly 6 years following the intervention [20]. Therefore, the nomogram developed in our study may be crucial for each patient in assessing the likelihood of recurrence for the next three and five years. This is the first nomogram to predict LRFS for patients with D-TSGCT in TMJ and we expect that it could offer help to clinical doctors in decision-making.

Our study has some limitations. Firstly, our nomogram is based on data from a single institution. It is necessary to have external validation. Secondly, all the patients underwent surgical resection as a treatment method, more treatment could be applied in the patients with a high risk of recurrence. Finally, the number of patients was small because of the scarcity, and further studies are needed to optimize a better prediction model.

## Conclusion

D-TSGCT of TMJ is accompanied by adjacent bone destruction. Tumors from the supra-articular cavity are more invasive and proliferative based on Ki-67 expression and clinical features. The first nomogram for predicting LRFS in patients with D-TSGCT was created in our study, this nomogram provides accurate calibration and discrimination.

### Electronic supplementary material

Below is the link to the electronic supplementary material.

Supplementary Material 1

## Data Availability

The datasets generated during the current study are available in the supplementary files.

## Abbreviations

D-TSGCT: Diffused-tenosynovial giant cell tumor
LRFS: Local recurrence-free survival
TMJ: Temporomandibular joint
CSF-1R: Colony-stimulating factor 1 receptor
