# Supplementary material for: Clinical retrospective analysis with a predictive model for diffused-tenosynovial giant cell tumors of the temporomandibular joint
Source: BMC Cancer. 2023 Nov 3;23:1062. doi: 10.1186/s12885-023-11587-7 (PMC10625237; doi:10.1186/s12885-023-11587-7)
Supplement: Supplementary file 1 — Supplementary Material 1 [file 12885_2023_11587_MOESM1_ESM.docx]

**Supplementary Table 1 Detailed information of the seventy cases with clinical characteristics**

| No. | name | sex | age | smoking | drinking | symptom | site（1super-articular cavity2subarticular cavity） | size | margin | local invasion（1Skullbase bone2Dura mater3Brain lobe4No） | condyle repair method | skull base repair method | condyle integrity | recurrence | post-operative complication |
| --- | --- | --- | --- | --- | --- | --- | --- | --- | --- | --- | --- | --- | --- | --- | --- |
| 1 | LDA | Male | 48 | Yes | Yes | H | 1 | 4.5*4.5 | negative | 1 | no | AF | Yes | Yes | No |
| 2 | YJB | Male | 59 | Yes | Yes | H，LMO | 1 | 2*2 | negative | 1 | no | TMF | No | No | No |
| 3 | SXZ | Female | 57 | No | No | FN | 1 | 3.5*3.7 | negative | 1 | no | No | Yes | No | No |
| 4 | LX | Female | 23 | No | No | H | 2 | 2*3 | negative | 4 | no | AF | No | No | No |
| 5 | XXF | Female | 39 | No | No | HL | 2 | 3.6*3 | negative | 4 | no | AF | No | No | No |
| 6 | TYF | Male | 32 | Yes | No | H，LMO | 1 | 3*6.1 | negative | 1 | costal cartilage | IB+TMF | No | No | No |
| 7 | SXA | Female | 50 | No | No | H | 2 | 3.6*2.5 | negative | 4 | no | AF | No | No | No |
| 8 | QY | Male | 41 | No | No | H，LMO | 2 | 2.6*1.7*1.8 | negative | 4 | no | AF | No | Yes | No |
| 9 | LZF | Female | 39 | No | No | H | 2 | 1.2*1.4 | negative | 4 | no | AF | Yes | No | H |
| 10 | DYX | Female | 37 | No | No | H | 1 | 6.1*4.8*3.5 | negative | 1 | no | No | Yes | Yes | No |
| 11 | SZQ | Male | 39 | No | No | HL | 1 | 4.8*3.6 | negative | 2 | no | No | Yes | No | No |
| 12 | JHX | Female | 38 | No | No | HL、FN | 1 | 3.7*4*3.5 | negative | 1 | no | TB | Yes | Yes | No |
| 13 | XHG | Male | 39 | No | No | M | 2 | 3*4 | negative | 4 | costal cartilage | TB | No | No | No |
| 14 | FCJ | Male | 58 | No | Yes | H | 1 | 2.0*1.5 | negative | 1 | no | TM | Yes | No | No |
| 15 | XXY | Male | 52 | Yes | No | HL | 1 | 4.8*3.7*4.2 | negative | 2 | no | TM+TMF | No | No | FP |
| 16 | HWJ | Male | 49 | Yes | No | HL | 1 | 5.6*4 | negative | 2 | no | TM+TMF | Yes | No | LMO、M |
| 17 | ZS | Female | 33 | No | No | H | 1 | 2.4*2.4*2.5 | negative | 1 | no | TM | No | No | No |
| 18 | GHX | Male | 26 | Yes | No | H | 2 | 3.5*3.2 | negative | 4 | costal cartilage | TM | No | No | No |
| 19 | YAY | Female | 57 | No | No | HL | 1 | 4.5*4.2*4.3 | negative | 1 | no | IB+SCMF | No | No | No |
| 20 | GFL | Female | 58 | No | No | H | 2 | 3.1*1.9*1.5 | negative | 4 | no | No | Yes | No | No |
| 21 | WWD | Male | 47 | No | Yes | PAM | 2 | 2.5*2.3 | negative | 4 | costal cartilage | No | No | No | No |
| 22 | LJJ | Male | 44 | No | No | LMO | 2 | 3.5*3.8 | negative | 4 | costal cartilage | No | No | No | No |
| 23 | DYJ | Female | 72 | No | No | PAM | 2 | 3.9*2.8 | negative | 4 | no | No | No | No | No |
| 24 | ZJZ | Male | 46 | No | No | M，HL | 1 | 5.6*4.6 | negative | 1 | no | No | Yes | No | No |
| 25 | DL | Male | 32 | No | No | HL | 1 | 4*3 | negative | 1 | no | BF | No | No | No |
| 26 | ZYH | Female | 26 | No | No | PAM | 2 | 1.1*0.4 | negative | 4 | no | AF | Yes | No | No |
| 27 | YYZ | Female | 55 | No | No | PAM | 1 | 2.9*1.6 | negative | 1 | no | AF | Yes | No | No |
| 28 | JHR | Male | 27 | No | No | H | 1 | 4*5*4.5 | negative | 1 | no | Medpor+AF | Yes | No | No |
| 29 | XCC | Female | 23 | No | No | LMO | 2 | 3.9*2.5 | negative | 4 | no | TMF | No | No | No |
| 30 | XJJ | Female | 50 | No | No | LMO | 1 | 3.5*3.5*2.8 | negative | 1 | no | TB | Yes | No | FP |
| 31 | XJJ | Female | 48 | No | No | PAM | 2 | 1.5*1.4 | negative | 4 | no | No | Yes | No | No |
| 32 | YQH | Male | 61 | No | No | LMO | 1 | 4*3 | negative | 1 | artificial joint | AF | No | No | No |
| 33 | JXJ | Male | 63 | Yes | Yes | PAM | 2 | 1.9*1.4 | negative | 4 | no | No | Yes | No | No |
| 34 | MHM | Female | 45 | No | No | H | 2 | 2.5*1.9*2 | negative | 4 | artificial joint | AF | No | No | FP |
| 35 | ZSL | Female | 24 | No | No | PAM | 1 | 3.7*2.6 | negative | 1 | artificial joint | No | No | Yes | No |
| 36 | CYS | Male | 56 | No | No | H | 1 | 3*5.6*2.7 | re-excision to obtain a negative margin | 2 | no | TB+TMF | Yes | No | M,FP,brain contusion |
| 37 | YTT | Female | 26 | No | No | H，PAM | 2 | 3.3*2.2 | negative | 4 | artificial joint | AF | No | No | 5 |
| 38 | YHF | Male | 27 | No | No | PAM | 1 | 5.7*3.4 | negative | 1 | no | No | Yes | No | No |
| 39 | MLF | Female | 53 | No | No | LMO | 2 | 2.9*1.9 | negative | 4 | artificial joint | No | Yes | No | 5 |
| 40 | WQW | Male | 48 | No | No | PAM | 2 | 2.7*1.9 | negative | 4 | artificial joint | No | No | No | No |
| 41 | TXH | Male | 47 | No | No | H，HL | 1 | 2.1*1.2 | negative | 1 | no | No | Yes | No | No |
| 42 | WWX | Female | 49 | No | No | H | 1 | 4.1*3.2 | negative | 1 | no | No | Yes | No | No |
| 43 | LXG | Male | 34 | No | No | PAM | 1 | 2*2.5 | negative | 1 | no | BF | Yes | No | No |
| 44 | HHJ | Male | 46 | No | No | HL | 1 | 6.2*4.1*6.5 | negative | 1 | artificial joint | No | No | No | S |
| 45 | TJH | Male | 48 | No | No | PAM | 2 | 2.1*1.5 | negative | 4 | no | No | Yes | No | No |
| 46 | ZXQ | Female | 59 | No | No | H，HL | 1 | 2.8*4*3.1 | negative | 1 | no | TM+BF | No | No | S |
| 47 | ZL | Male | 39 | Yes | No | PAM | 1 | 4.7*3.6*5.1 | negative | 2 | no | TM+TMF | Yes | No | LMO |
| 48 | WGY | Male | 26 | No | No | H | 1 | 3.9*4.3*2.5 | negative | 2 | no | TM+TMF | Yes | Yes | LMO |
| 49 | FQ | Male | 32 | No | No | PAM | 1 | 4.9*3.7 | negative | 2 | no | TM+TMF | Yes | Yes | No |
| 50 | WSC | Male | 50 | No | No | HL | 1 | 5*3*4 | negative | 3 | no | TM+TMF | No | No | FP |
| 51 | LJJ | Male | 43 | No | No | H | 1 | 2.1*3.3*4 | negative | 3 | no | TM+ALTF | No | Yes | M |
| 52 | HHL | Male | 32 | No | No | H | 1 | 4.5*3 | negative | 2 | no | TB+TMF | No | No | H |
| 53 | MXP | Male | 46 | No | No | H | 1 | 3*4.8 | negative | 2 | no | TB+TMF | Yes | Yes | No |
| 54 | XJ | Male | 26 | No | No | H | 1 | 4*5*4.5 | negative | 2 | no | TM | Yes | No | No |
| 55 | YJS | Male | 54 | No | No | HL | 1 | 1.7*1.2 | negative | 2 | no | TB+TMF | Yes | No | HL、FP |
| 56 | MRJ | Female | 52 | No | No | H、HL | 1 | 2.9*2.4 | negative | 1 | no | TB | Yes | No | No |
| 57 | MCX | Female | 48 | No | No | H | 1 | 3.8*3.3 | negative | 2 | no | TM | Yes | No | No |
| 58 | ZZL | Female | 50 | No | No | H、LMO | 1 | 0.6*0.4*0.2 | negative | 2 | no | TM+TMF | No | No | LMO |
| 59 | WLC | Male | 42 | No | No | HL | 1 | 3*4*1 | negative | 1 | no | TM | Yes | No | No |
| 60 | ZJY | Male | 47 | Yes | Yes | HL | 1 | 3.8*2.5*2.1 | negative | 2 | no | TB+TMF | Yes | No | HL |
| 61 | XJ | Female | 32 | No | No | H | 1 | 4.7*3.8 | negative | 2 | no | TM+TMF | Yes | No | HL |
| 62 | MHZ | Female | 46 | No | No | H | 1 | 2.6*2.8*2.4 | negative | 2 | no | TM | Yes | Yes | FP |
| 63 | LXJ | Female | 65 | No | No | H | 1 | 5.9*3.7*3.6 | negative | 1 | no | TM+ALTF | No | No | FP |
| 64 | LBH | Male | 52 | No | No | H | 1 | 1.2*2 | negative | 1 | no | TM+TMF | Yes | No | S |
| 65 | ZFY | Female | 30 | No | No | LMO | 1 | 4*3 | negative | 1 | costal cartilage | TM+BF | No | No | No |
| 66 | CZQ | Male | 36 | No | No | HL | 1 | 2.8*2.7 | negative | 2 | no | TM+ALTF | No | Yes | FP |
| 67 | ZCF | Female | 62 | No | No | LMO | 1 | 5.3*3 | negative | 1 | no | TM+TMF | Yes | No | FP |
| 68 | ZBB | Male | 28 | No | No | H.LMO | 2 | 3*3 | negative | 4 | artificial joint | No | Yes | No | No |
| 69 | CZH | Male | 26 | No | No | PAM | 2 | 4*4 | negative | 4 | artificial joint | No | Yes | No | No |
| 70 | LJM | Female | 53 | No | No | PAM | 1 | 4*4.5 | negative | 1 | no | TMF | No | No | No |

Note: Note: FN=facial numbness, M=Malocclusion, S=swelling, FP=facial paralysis, H=Headache, HL=hearing loss, LMO=limited mouth opening, P=pain, PAM=pre-auricular mass, SCMF=sternocleidomastoid flap, TMF=temporalis muscle flap, BF=belly fat, ALTF=anterolateral thigh flap, AF=adjacent flap, TB=temporal bone, IB=iliac bone, TM=titanium mesh


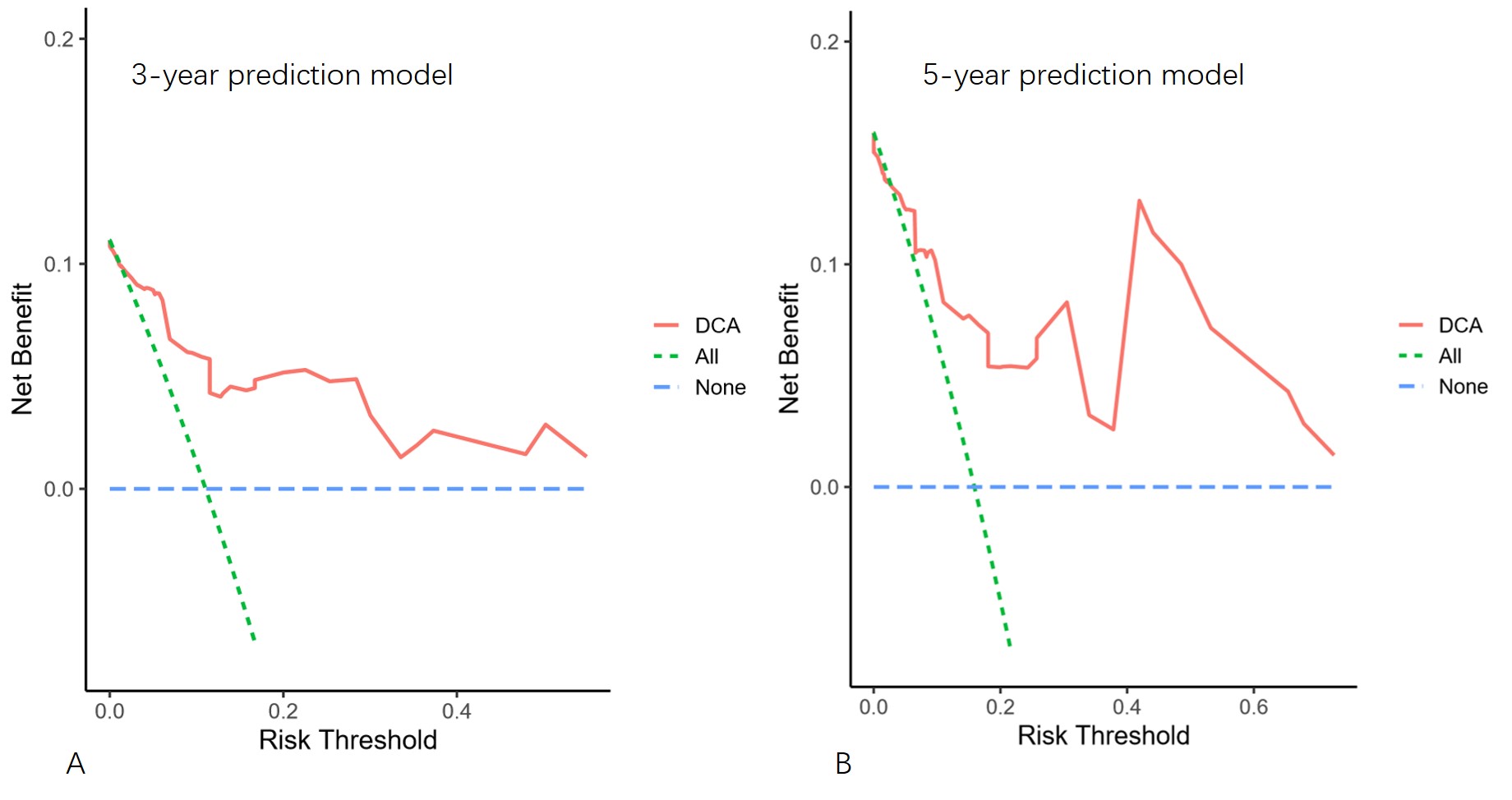


Supplementary Fig 1 Decision curve analysis for the nomogram model in the training cohort. (A) 3-year LRFS; (B) 5-year LRFS.
